# Auto-Reactive Th17-Cells Trigger Obsessive-Compulsive-Disorder Like Behavior in Mice With Experimental Autoimmune Encephalomyelitis

**DOI:** 10.3389/fimmu.2018.02508

**Published:** 2018-10-31

**Authors:** Ravi Kant, Shweta Pasi, Avadhesha Surolia

**Affiliations:** ^1^Molecular Science Laboratory, National Institute of Immunology, New Delhi, India; ^2^Molecular Biophysics Unit, Indian Institute of Science, Bangalore, India

**Keywords:** Th17, autoimmune, encephalomyelitis, behavior, grooming

## Abstract

Th17-lymphocytes are well known for their deleterious role in autoimmunity. But does the notoriety of this repertoire extend beyond autoimmunity? In the present study we employed experimental autoimmune encephalomyelitis as model system to study the role auto-reactive Th17 cells in neuropsychiatric disorders. The mice with experimental autoimmune encephalomyelitis exhibited exaggerated grooming activity. The observed behavioral anomaly resembled obsessive compulsive disorder (OCD) upon analysis of grooming microstructure, induced grooming, marble burying and nestlet shredding. The observed OCD like behavior was relieved upon Th17 cell depletion; alternatively, it could alone be induced by adoptive transfer of myelin oligodendrocyte glycoprotein (35-55) reactive Th17 in *B6.Rag1*^−/−^ mice. The observed OCD like behavior was also alleviated upon treatment with a selective serotonin reuptake inhibitor, fluoxetine.

## Introduction

Obsessive compulsive disorder (OCD), a component of anxiety disorder spectrum, is hallmarked by intrusive, recurring thoughts and anxiety-driven compulsive behaviors that act as “stress-relievers” for the sufferer. OCD has a global prevalence of ≥2% (1). Despite, extensive experimental efforts in understanding the pathogenic mechanisms of the disease, its association with immune system, in general, and autoimmunity, in particular, remains poorly defined. While previous attempts have been largely aimed at determining the levels of various cytokines or immune-cell populations in OCD patients which appear more as an effect rather than provocative cause of the illness. Though, there are reports that document the co-existence of OCD and autoimmunity, such as in pediatric autoimmune neuropsychiatric disorders associated with streptococcal infections (PANDAS, a subset of OCD) (2), multiple sclerosis (MS) (3–6), inflammatory bowel disease (IBD) (7), systemic lupus erythematosus (SLE) (8) and to some extent in rheumatoid arthritis (RA) (9), where some positive correlation with OCD has been found; the underlying mechanisms still remains elusive.

Alterations in the Th17 cell population and the cytokines that govern their differentiation such as IL-6, have also been reported in a number of neuropsychiatric illnesses. For example, IL-6 levels are elevated in a subset of patients with depression (10–13). CD4^+^ T-cells isolated from patients with generalized anxiety disorder show an enhanced Th17 phenotype upon activation (14, 15). Interestingly, in some cases it has been observed that Th17 cells are alone capable of inducing depression-like behavior in rodents (16). Thus, potential of Th17 cells to alter functioning of central nervous system (CNS) warrants investigations in chronic diseases concurrent with neuropsychiatric illnesses.

The role of Th17 cells in MS and its animal model i.e. experimental autoimmune encephalomyelitis (EAE) is well studied. Initially believed to be mediated by Th1 cells, there has been a paradigm shift in our understanding of its pathogenesis over the years with numerous lines of evidences highlighting the role of Th17 cells in the disease. For instance, autoreactive Th17 cells can migrate efficiently across the blood brain barrier (BBB) aided by the cytokines such as IL-17 and IL-22, and cell surface receptor CCR6 (17, 18). Further, upon entry into the central nervous system (CNS), Th17 cells orchestrate neurodegeneration through several mechanisms (19–21). Adoptive transfer experiments with Th17 cells and studies in IL23p19, IFNγ and IL-17 deficient mice have demonstrated that the Th17 cell subset is more important than the Th1 subset for the induction of EAE (22–25). The role of Th17 cells is also being appreciated in MS. MS patients are reported to have high number of Th17 cells in their circulation and CNS (26, 27). CSF concentrations of IL-17 are also significantly elevated in MS patients than healthy controls (28). Microarray analysis of acute or chronic MS has revealed upregulation of IL-17 mRNA in both acute and chronic active MS lesions (26, 29).

A number of reports have revealed concurrence of MS with neuropsychiatric disorders such as OCD (3–6), depression (4, 30–32), bipolar disorder and psychosis (32, 33). MS pathology is characterized by aberrant Th17 responses which may enhance vulnerability of the MS affected individuals to neuropsychiatric illnesses. Alternatively, increase in Th17 cell population in MS may underlie the psychiatric manifestations associated with the disease.

In the present study, we employed chronic experimental autoimmune encephalomyelitis (cEAE) as a model system to characterize the psychological abnormalities associated with MS. The mice with cEAE exhibited excessive grooming activity concomitant with an increase in CNS infiltrating Th17 cells. The specific contribution of Th17 cells in the observed behavioral anomaly was determined by employing model systems wherein Th1 or Th17 cell repertoires were selectively depleted or reconstituted. Th17 cells but not Th1 cells were noted to trigger repetitive behavior in mice besides autoimmunity. The observed compulsive behavior was characterized by analyzing the grooming syntax, nestlet shredding and marble burying. It was noted to resemble OCD, a neuropsychiatic illness prevalent in humans and was alleviated upon treatment with fluoxetine (a selective serotonin reuptake inhibitor).

## Materials and methods

### Mice

*SJL/J, C57BL/6J, B6.Rag1*^−/−^, *B6.2D2, B6.IFN*γ^−/−^ mice procured from Jackson Laboratories were housed under standard housing conditions on a 14-h light and 10-h dark cycle, and acclimatized for at least 1 week before conducting experiments. All animals received *ad libitum* access to food and water. All experiments were performed in accordance with the guidelines of the Institutional Animal Ethics Committee (IAEC) of The National Institute of Immunology, New Delhi, India.

### Treatments

Fluoxetine hydrochloride (Tocris Bioscience, USA) was reconstituted in phosphate buffered saline (PBS). Digoxin (Sigma, USA) was dissolved in dimethyl sulfoxide and further diluted appropriately in PBS to get the desired concentration at the time of injection. Peptides viz. MOG(35-55), PLP(131-151) acquired from Anaspec, USA used for disease induction were dissolved in PBS and emulsified in Freund's complete adjuvant (Sigma, USA). Antibodies anti-CD3, anti-CD4, PE-anti-TCRβ, APC-anti-IFNγ, FITC-anti-IL-17A, anti-IL-4, anti-IL-23, Alexa Fluor 488-anti-rat, Alexa Fluor 555-anti-rabbit, cytokines, IL-12, IL-17A, IL-21, IL-23, TGFβ, IL-6 were purchased from BD Pharmingen, Abcam, Cell Signaling Technology, and RnD Technologies. Dulbecco's Modified Eagle Medium (DMEM) and fetal bovine serum (certified) were procured from Gibco, USA.

### Induction and assessment of autoimmune encephalomyelitis (EAE)

EAE was induced in *C57BL6/J* or *IFN*γ^−/−^ mice (female, 8–10 week old) by subcutaneous immunization with MOG (35-55) (100 μg emulsified in Freund's complete adjuvant, CFA); intra-peritoneal injections of Pertussis toxin (200 ng, Sigma, USA) were given on day 0 and 2. For induction of relapsing remitting form of EAE *SJL/J* mice (female, 8–10 week old) were immunized with PLP (131-151) (100 μg emulsified in CFA). Disease assessment following onset was done as previously described (34–36). Briefly, clinical disability in case of EAE with classical signs was scored on a scale of 0–5, where, 0, no detectable signs of EAE; 1, complete tail paralysis; 2, wobbly gait; 3, complete hind limb paralysis; 4, complete hind and fore limb paralysis or moribund; 5, dead. Whereas, in EAE with atypical signs, clinical disability was scored as follows, 0, no detectable signs; 1, tail paralysis, hunched appearance, unsteady walk; 2, ataxia, head tilt, hypersensitivity; 3, severe ataxia, spasticity or knuckling, severe proprioception defects; 4, moribund and 5, dead.

### Behavioral analysis

*Self-Grooming:* The mice were housed individually in transparent polycarbonate cages, acclimated for about 2 weeks, before the start of experiment. Behavioral activities were recorded under mild-red illumination using camcorders with improved night vision (Sony). Grooming behavior was analyzed in detail as described previously (37, 38). Briefly, grooming behavior was analyzed as follows: any grooming activity, containing majority of grooming sequences, lasting for more than 10 s with a pause of not more than 6 s was considered as a grooming bout. When the pause during grooming transitions was more than 6 s the bout/transition was considered interrupted. Correct transitions include: 0-1, 1-2, 2-3, 3-4, 4-5, 5-0, where 0, no grooming; 1, paw-licking; 2, nose/face/head wash; 3, body grooming; 4, leg licking; 5, tail/genital grooming. Any transitions other than those mentioned above were considered incorrect. *Induced-grooming:* The test mice (diseased/healthy) were placed in empty transparent polycarbonate cages, following 10 min of acclimatization, the animals were lightly misted with water in the facial region, and grooming-activities were recorded and analyzed for a period of 15 min on 5 consecutive days. *Marble burying:* Marble burying test was performed as described previously (39). Briefly, the test cage (27 × 17 × 11 cm) was prepared by placing 20 glass marbles (1 cm diameter, autoclaved) evenly on bedding material (saw dust, 4–5 cm thick). The experimental animal was left undisturbed for 15 min in the test cage in an isolated place. A marble was considered buried when >90% was covered in bedding material. *Nest building/nestlet shredding:* Mice were placed in a cage with two cotton nestlets for 12 h. The quality of the nest built was scored on a scale of 0–5, where 0 signified untouched nestlet and 5 signified complete nest with roof. Partially built nests were scored as 1, 2, and 3 depending on height of the nest walls (40). Nestlet shredding was quantified in terms of percent dry weight (of nestlet) left after 3 h. *Thermal analgesia:* Response to thermal stimuli was analyzed as described previously (41). Briefly, mice were placed on hot plate (50–52°C). The latency to the first hind paw licking or withdrawal was recorded as a measure of nociceptive threshold. A cut-off of 60 s was set up to avoid burn injury. *Mechanical allodynia:* The response to mechanical stimuli was measured as described previously (42) using electronic von Frey instrument (IITC Inc., USA). Briefly, test animal was placed inside Polymethyl methacrylate (PMMA) housing set on mesh floor stand 30 min before the start of the measurements. The mechanical stimulus was applied to the middle of plantar surface of right hind-paw using rigid polypropylene tips mounted on von Frey probe. The maximum amount of pressure (in terms of grams) that led to paw withdrawal response (paw retraction, licking, jumping) was recorded.

### Isolation of CNS derived mononuclear cells, intracellular staining and FACS analysis

The mononuclear cells were harvested from brain tissue and analyzed as described previously (43, 44). Briefly, mice were perfused intra-cardially with ice-cold PBS, brain tissue was dissected-out and homogenized. The mononuclear cells were isolated from brain tissue homogenate using 30/70% percoll (Sigma, USA) gradient. The mononuclear cells thus obtained were stimulated with PMA (10 nM, Sigma, USA) and Ionomycin (1 nM, Sigma, USA) for 4–5 h in the presence of Golgi plug (BD Pharmingen, USA). The cells were harvested and incubated with of Fc-block (1 μg/million cells, BD Pharmingen, USA) in FACS staining buffer (BD Pharmingen, USA) for 15 min on ice. Thereafter, cells were washed and incubated with anti-mouse TCRβ (PE) for 30 min at room temperature. Subsequently, for intracellular staining, cells were resuspended in cytofix/cytoperm buffer (BD Pharmingen, USA) for 15 min at room temperature, washed with perm/wash buffer (BD Pharmingen, USA) and incubated with anti-mouse IL-17A (FITC), anti-mouse IFNγ (APC) for 30 min on ice. Following this, cells were washed and resuspended in FACS staining buffer containing 7-AAD. Samples were acquired immediately on BD FACS Calibur and analyzed using Flowing software. Analysis was performed on live single cell population gated from FSC vs. SSC plots.

### T cell differentiation and adoptive transfer

Differentiation of naïve T-cells into MOG (35-55) reactive Th17 or Th1 cells was carried out as described in an earlier report (19). Briefly, splenocytes isolated from *B6.2D2* mice were magnetically sorted (MACS, Miltenyi Biotech, Germany) for CD4^+^ CD62L^+^ T-cells which were then cultured in either Th1 or Th17 polarizing conditions. For Th1 polarization, naive CD4^+^ T-cells were cultured with CD90^+^ depleted irradiated splenocytes in the presence of MOG (35-55) 25 μg/ml each, IL-12 (10 ng/ml), anti-IL-4 (20 μg/ml) while for Th17 polarization, CD4^+^ CD62L^+^ T cells were cultured with CD90^+^ depleted irradiated splenocytes in the presence of MOG (35-55) 12.5 μg/ml each, TGFβ (3 ng/ml), anti-IL-23 (20 ng/ml) and IL-6 (20 ng/ml). Cells were re-stimulated every week and harvested after 3 days of second re-stimulation. For adoptive transfer experiments, MOG (35-55 reactive Th1 cells or Th17 cells, enriched to a purity of >90% (employing kits provided by Miltenyi Biotech, Germany) were transferred (5–10 million) intravenously into *B6.Rag1*^−/−^.

### Immunohistochemistry

The mice at an experimental time point were perfused trans-cardially with ice cold PBS (30 ml). Intact brain-tissue was dissected-out and flash-frozen for cryo-sectioning. Immuno-histochemical staining of tissue sections (10 μm) was performed as described previously (45). Briefly, frozen tissue-sections were fixed, permeabilized using acetone-methanol mixture at −20°C for 10 min. The tissue-sections were treated with antibody diluent (Cell Signaling Technology, USA) for 1 h at room temperature to block non-specific antigen binding sites. Thereafter, tissue-sections were incubated with primary antibodies (anti-CD4, anti-IL-17A, anti-IFNγ, diluted in antibody diluent) at 4°C for 16–18 h. Subsequently, tissue-sections were washed thoroughly with PBST (PBS + 0.05% Tween-20) and incubated with secondary antibodies (Alexa Fluor 488-anti–rat, Alexa Fluor 555-anti–rabbit) at 37°C for 1 h. After incubation with secondary antibodies tissue-sections were washed and mounted using Fluoroshield-DAPI (Sigma, USA). Images were acquired at 2 or 20X magnifications in a microscope equipped with a motorized stage (Ziess Axio Imager 2) and images were processed using Image J software.

### Neurotransmitters, cytokines quantification

Intact brain-tissue was dissected-out after rapid decapitation. Brains were placed in RNA later (Sigma, USA) for 24–48 h at 4°C. Various anatomical regions of the brain tissue were dissected under microscope. Thereafter, 10% (w/v) homogenates were prepared in buffer containing protease inhibitors (Abcam, USA). For analysis, samples were normalized for total protein content, levels of neurotransmitters (viz. serotonin, glutamate, dopamine), cytokines (viz. IFNγ, IL-17A) were estimated in tissue homogenates using commercially purchased kits from BioVision Inc., USA and ELISA based kits provided by Ebioscience, USA respectively. Observed levels were normalized to control (medium or homogenization buffer).

### Statistical analysis

Analysis of variance (ANOVA, single factor) or student's *t*-test (unpaired, two tailed) were employed for multiple and binary comparisons respectively. Differences in mean with *p*-value less than 0.05 were considered significant.

## Results

### Mice with chronic autoimmune encephalomyelitis exhibit repetitive behavior

**Figure 1:**
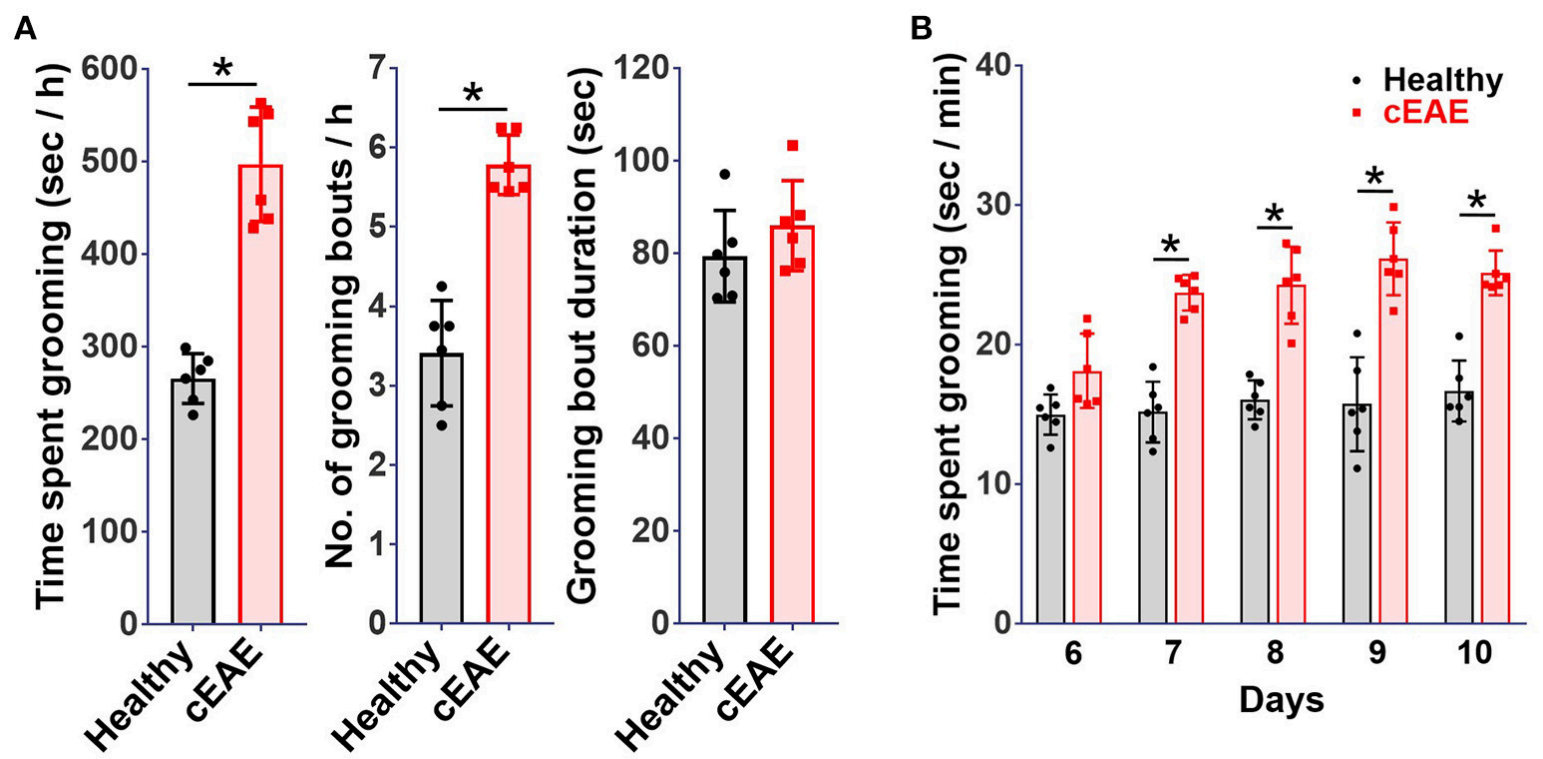
Compulsive-grooming behavioral phenotype accompanies chronic autoimmune encephalomyelitis (cEAE). Chronic autoimmune encephalomyelitis (cEAE) was induced in *C57BL6/J* mice (female, 8-10 week old) by injecting MOG(35-55), **(A)** grooming analysis *viz*. time spent grooming, number of grooming bouts, duration of grooming bouts (on day 10). For induced grooming analysis mice with cEAE were misted with water (facial region) and analyzed for grooming activity for 15 min from day 6 to 9, **(B)** time spent grooming. mean ± S.D., *n* = 6, ^*^p < 0.05, data representing three to four independent experiments.

**Figure 2:**
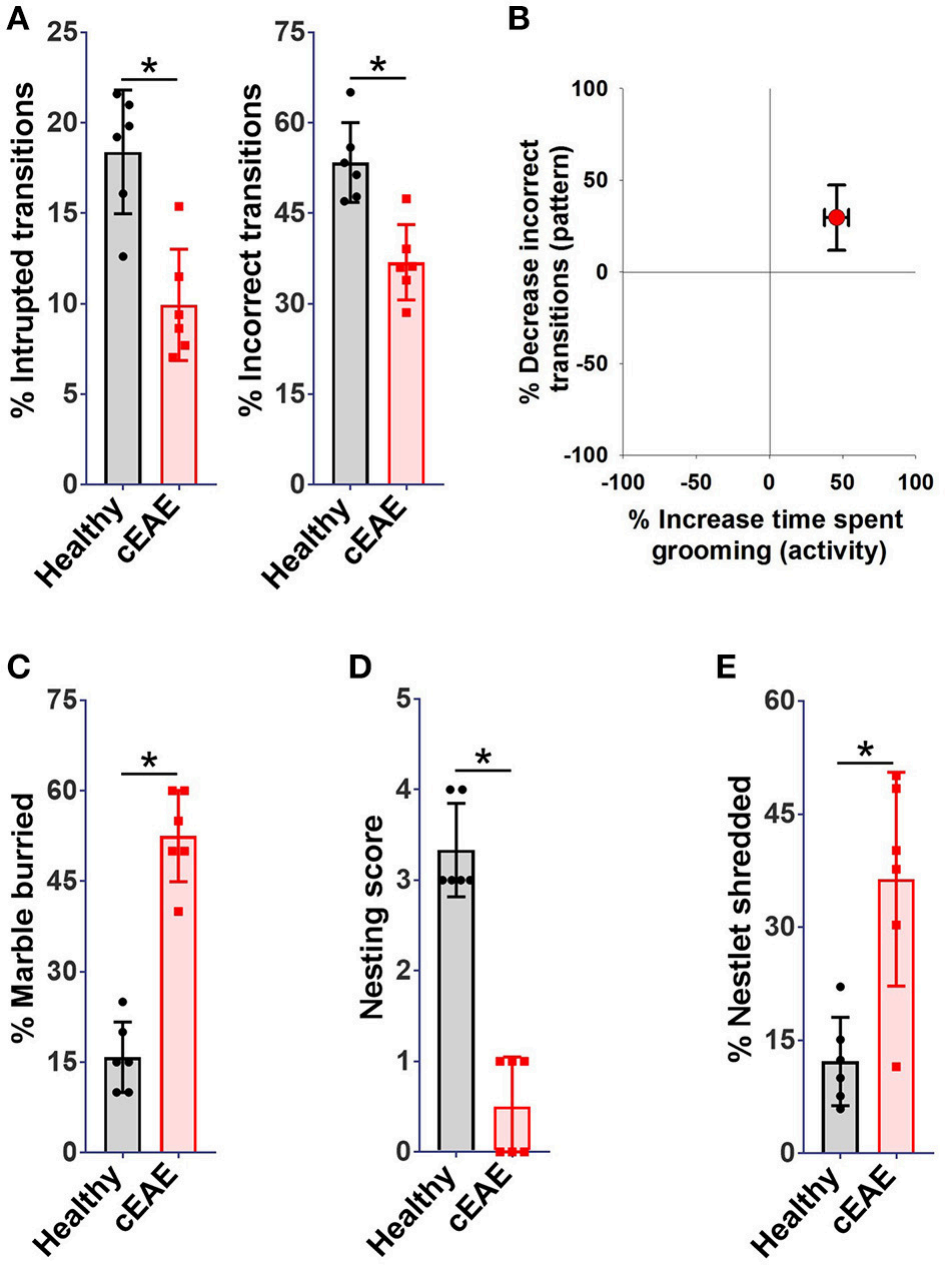
Repetitive-behavioral phenotype observed in mice with cEAE resembles obsessive-compulsive disorder - a pathological neuropsychiatric phenotype prevalent in humans. *C57BL6/J* mice (female, 8-10 week old) immunized with MOG (35–55) were analyzed for grooming activity and syntax (day 10), **(A)** percent interrupted, incorrect transitions, **(B)** correlation between activity (times spent grooming) and pattern (percent incorrect transitions). *C57BL6/J* mice (female, 8–10 week old) immunized with MOG (35–55) were analyzed for nestlet shredding, nesting and marble burying behavior (day 10), **(C)** percent marble buried, **(D)** nesting score, **(E)** percent nestlet shredded. mean ± S.D., *n* = 6, ^*^p < 0.05, data representing three independent experiments.

Chronic experimental autoimmune encephalomyelitis (cEAE) was induced in *C57BL6/J* mice employing MOG (35-55). The immunized mice were housed individually and their behavioral activities were video-recorded on day 10 post-immunization. The animals were noted to be most active between 8 p.m. and 12 p.m. in consent with published reports (37). Hence, components of routine animal behavior namely eating, drinking, walking, sleeping, grooming, etc. were video-scrutinized during that time frame only. The video-recordings were analyzed in a double-blind manner. A comparison was drawn between the routine activities of immunized mice and healthy control mice housed under identical conditions. Mice in both experimental groups *viz*. cEAE or healthy control displayed comparable pattern in execution of all the innate behaviors except grooming. Mice with cEAE were noted to devote, on an average, 60-70% more time in grooming themselves *vis-à-vis* their healthy counterparts, evident even before the clinical onset of EAE (Figure 1A). The symptoms of EAE appeared on day 12-14 post-immunization (Supplementary Figure 1). The number of grooming bouts were noted to be significantly increased that contributed to the observed difference in total time spent grooming (Figure 1A). On few occasions, the diseased mice were also noted to develop peculiar body patterns such as loss of facial hair/whiskers, balding (typically involving the ventral regions of the body), self-inflicted injuries, 8–10 weeks post-immunization (Supplementary Figure 2). Association of compulsive grooming with cEAE was further confirmed by artificially stimulating it by misting facial region with water. It was noted that cEAE-mice spent about 50–70% more time grooming themselves than that observed in their healthy counterparts (Figure 1B). Thus, the results of induced grooming analysis correlated well with those of detailed behavioral studies. Expectedly, the induced grooming bouts were mostly restricted to the facial (head) region. The diseased mice displayed normal response to mechanical (von Frey test) and thermal stimuli (hot plate test), establishing normalcy of sensory-innervations (Supplementary Figure 3). Thus, the plausibility of exaggerated grooming as a result of altered nociception was ruled out. To further characterize the observed behavioral anomaly the grooming microstructure was analyzed in detail. While executing a grooming bout the affected mice followed the cephalocaudal (from head to tail) syntax with relatively more time devoted to face / head grooming (phase 2/3 of the grooming chain) (Supplementary Movies 1, 2). More importantly, detailed analysis of the grooming microstructure revealed a significant decrease in incorrect and interrupted transitions (Figures 2A,B). In simple terms, the diseased mice groomed excessively while maintaining grooming-syntax, a hallmark of obsessive compulsive disorder (46, 47). Further the animals were subjected to marble burying and nesting/nestlet shredding test. While the healthy mice buried only 20–30% of marbles in a given time period, the cEAE mice buried 50–60% of glass marbles (Figure 2C). Although, there was a marked increase in nestlet shredding behavior but nest building was severely compromised (Figures 2D,E). This marked increase in compulsive burying and nestlet shredding behavior further suggests its similarity to OCD and defective nesting behavior signifies cognition defect.

In order to delineate whether the observed behavioral abnormalities extend to a related EAE model; explorations in relapsing-remitting form of EAE (rrEAE) were carried out. rrEAE was induced in *SJL/J* mice by immunization with PLP(131-151). Most of the mice immunized with PLP(131-151) displayed clinical onset of disease on day 12–14 (Supplementary Figure 1). Unexpectedly, the differences in grooming activities of diseased (*viz*. those with rrEAE) and their healthy counterparts were insignificant when analyzed *in-situ* on day 10 post-immunization. Also, diseased animals did not show any signs of pathological hair removal during the entire span i.e., 8–10 weeks, of our study (Supplementary Figure 4). Hence, the exaggerated grooming activity was found restricted to mice afflicted with MOG (35-55) induced chronic EAE.

### Auto-reactive Th17 cells drive pathological grooming in mice

**Figure 3:**
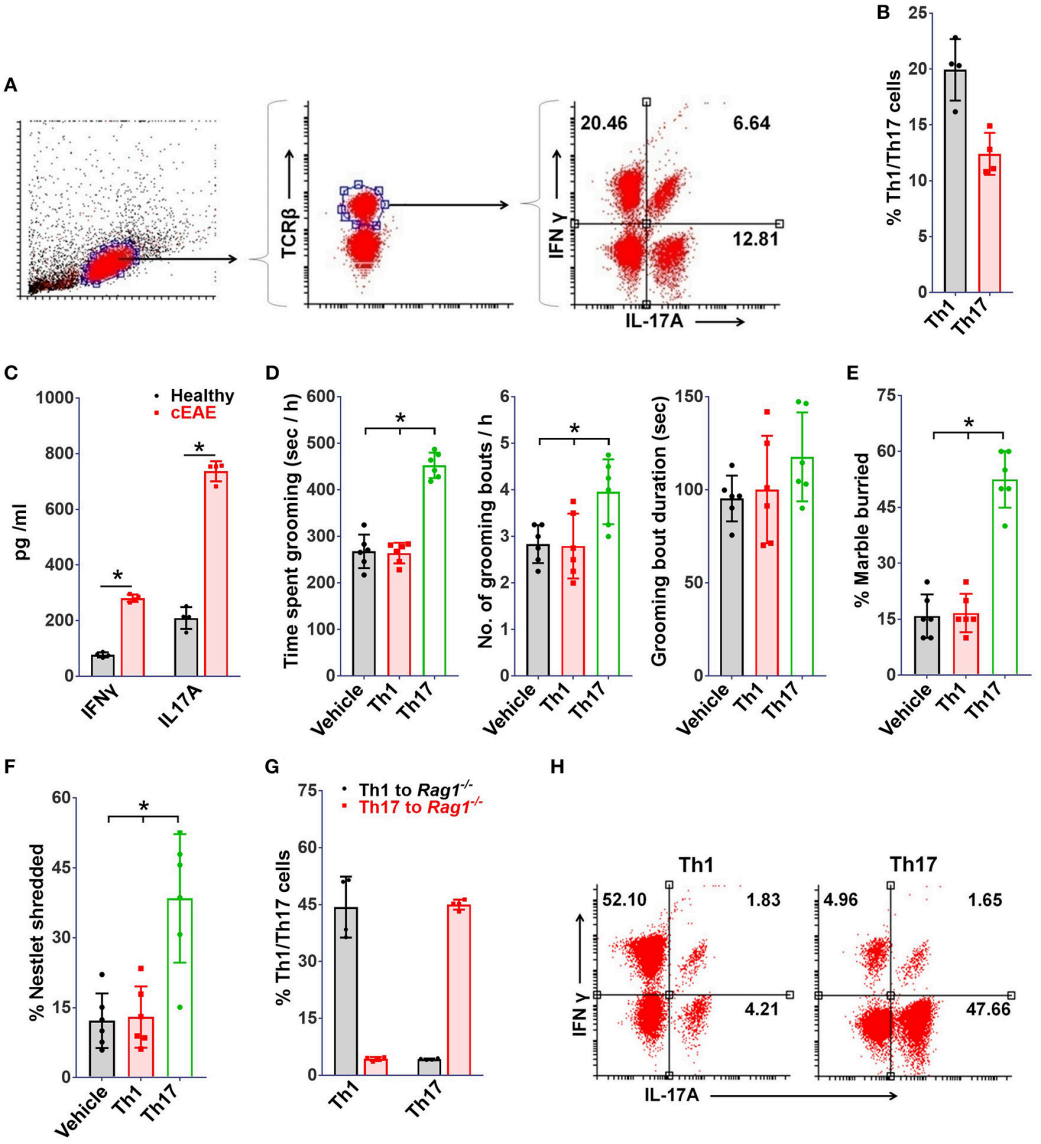
Th17 but not Th1-cells drive grooming pathology. To study immune cell infiltration, brain derived mononuclear cells (MNCs) were isolated and analyzed (on day 10 post-immunization) for Th1, Th17 cell populations by FACS, **(A)** representative FACS dot plots, **(B)** percent TCRβ^+^ IFNγ^+^ (Th1) and TCRβ^+^IL-17A^+^ (Th17) cells (*n* = 4). **(C)** Levels of IFNγ and IL-17A in brain homogenates (*n* = 4). *Rag1*^−/−^mice (8-10 week old, female) were transferred with Th1 or Th17 cells derived from *2D2* mice, **(D)** grooming analysis *viz*. time spent grooming, number of grooming bouts, duration of grooming bout on day 10 post-transfer (*n* = 6), **(E)** percent marble buried, **(F)** percent nestlet shredded; immuno-phenotype of adoptively transferred Th1/Th17 cells (on day 10 post-immunization), **(G)** percent Th1 or Th17 cells (*n* = 4), **(H)** representative FACS dot plots. mean ± S.D., ^*^p < 0.05.

Given the central role of Th1 and Th17-lymphocytes in EAE, identification of the precise immunological trigger for the behavioral pathology was warranted. For a comprehensive analysis, brain derived mononuclear cells (MNCs) were isolated and characterized by flowcytometry on day 10 post-immunization. Brain-tissue of mice with cEAE yielded a total of 0.3–0.5 million cells. Among the CD4^+^ cells isolated, 18 ± 4%, 15 ± 6% were Th1 and Th17 respectively (Figures 3A,B). Also, the levels of inflammatory cytokines associated with Th1, Th17 phenotype i.e. IFN-γ, IL-17A were significantly increased (Figure 3C), indicating their possible involvement in grooming elicit.

**Figure 4:**
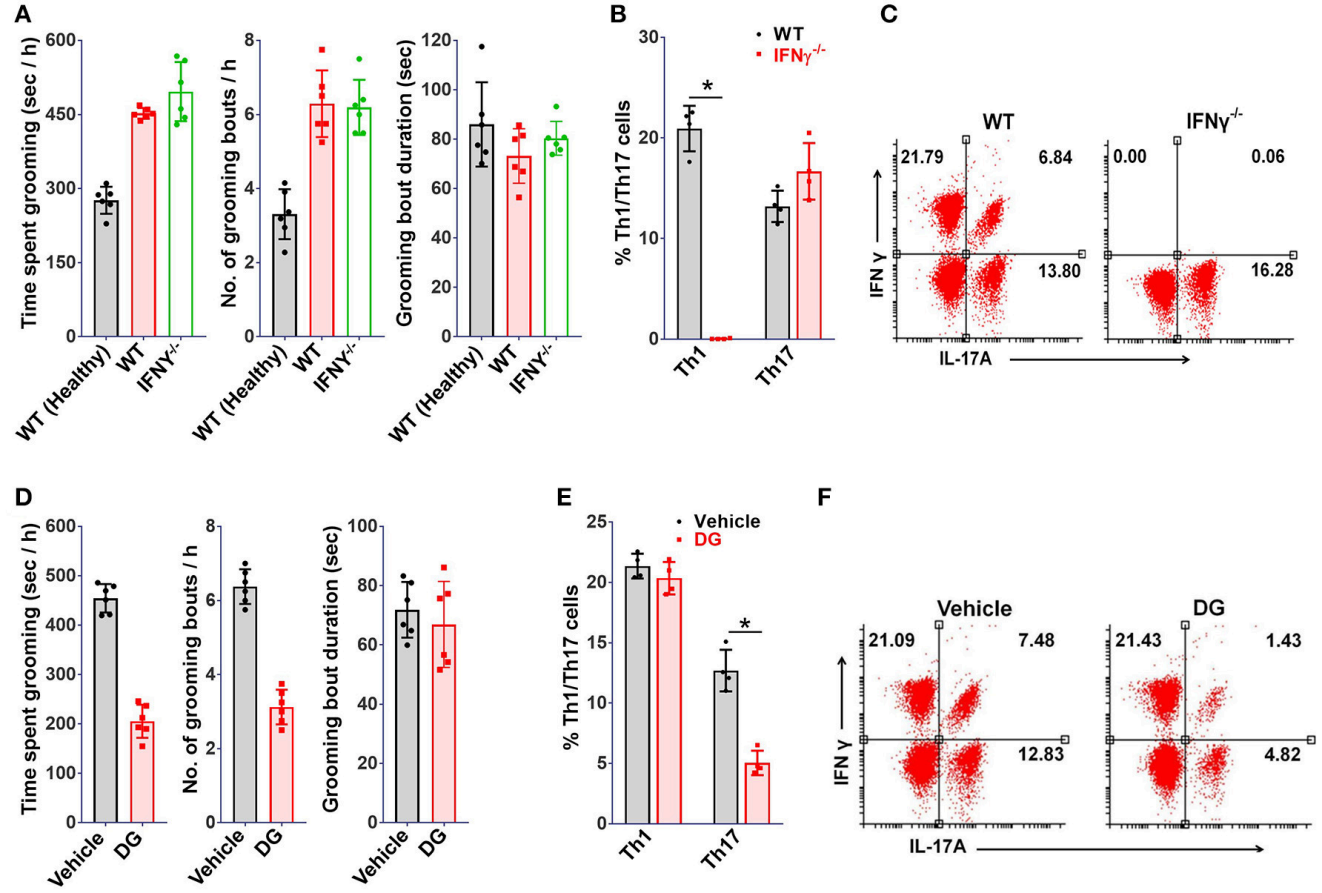
Th17 depletion ameliorates grooming pathology. *IFN*γ^−/−^ or wild type (WT) mice (8-10 week old, female) were immunized with MOG(35-55), **(A)** grooming analysis *i.e*. time spent grooming, number and duration of grooming bouts (on day 10 post-immunization) (*n* = 6), proportions of Th1 or Th17 cells in mononuclear cells derived from brains of MOG(35-55) immunized *IFN*γ^−/−^ mice (on day 10 post-immunization), **(B)** percent Th1 or Th17 cells (*n* = 4), **(C)** representative FACS dot plots. Mice (WT) immunized with MOG(35-55) injected intraperitoneally with digoxin (DG, 0.5 mg/kg body weight) from day 4-9, **(D)** grooming analysis *viz*. time spent grooming, number and duration of grooming bouts (on day 10) (*n* = 6), **(E)** percent Th1 or Th17 cells (on day 10 post-immunization) (*n* = 4), **(F)** representative FACS dot plots. mean ± S.D., ^*^p < 0.05.

Further, to determine the relative contribution of Th1 or Th17 cells toward development of the observed grooming pathology, MOG (35-55) specific Th1 or Th17 cells derived from *B6.2D2* mice (*2D2*, transgenic mice expressing MOG (35-55) specific T-cell receptor) were adoptively transferred into *B6.Rag1*^−/−^ (*Rag1*^−/−^) mice. As expected, mice infused with either Th1 or Th17 cells developed signs of autoimmune encephalomyelitis (12–14 days post-immunization) (Supplementary Figure 1). Although mice in both experimental groups developed signs of EAE to our surprise only the mice which were transferred with Th17 cells showed significant differences in grooming activity on day 10 post-transfer (Figure 3D). Also, a concomitant increase in marble burying and nestlet shredding behavior was noted (Figures 3E,F). Additionally, the immuno-phenotype of Th1 or Th17-cells transferred adoptively changed minimally when analyzed in brain derived MNCs subsequent to behavioral analysis (day 10 post-immunization), thereby assuring that the phenotype of infused lymphocytes was retained (Figures 3G,H). To ascertain the specific association of pathological grooming with Th17-cells, cEAE was induced in *B6*.*IFN*γ^−/−^ (*IFN*γ^−/−^) mice. Both wild type *(WT)* and *IFN*γ^−/−^ mice displayed signs of EAE (Supplementary Figure 1). EAE observed in case of *IFN*γ^−/−^ mice had a symptomatic similarity to that of its atypical form. A significant increase in grooming activity was seen in both *IFN*γ^−/−^ and wild type mice exhibiting EAE *vis-à-vis* healthy controls (wild type), when examined on day 10 post-immunization. However, the severity of compulsive grooming in *IFN*γ^−/−^ and diseased C57BL6/J mice remained comparable (Figure 4A). Also, relatively higher frequencies of Th17-cells were noted in brain derived MNCs of *IFN*γ^−/−^ mice isolated after behavioral analysis which may be attributed to the lack of inhibition imposed by IFNγ^+^ Th1-cells on Th17-cell differentiation (Figures 4B,C). Since autoimmunity in *IFN*γ^−/−^ mice is characterized by deficiency of the encephalitogenic Th1 repertoire (48) and is predominantly Th17-cell driven, the role of Th17-cells is hence further substantiated.

As of now it was obvious that Th17-cells were responsible for the compulsive behavior. Hence, it could be hypothesized that molecules affecting Th17 differentiation should relieve the behavioral compulsions. To test this, mice affected with cEAE were treated with a selective inhibitor of Th17 differentiation i.e. digoxin (DG) (49). In agreement with our hypothesis, the grooming activity in mice immunized with MOG (35-55) was reduced by 40–50% following DG treatment (Figure 4D). Simultaneously, analysis of brain derived MNCs revealed a selective reduction in Th17-cellular infiltrates with minimal alterations in the Th1 pool (Figures 4E,F). The observations thus further confirmed an intimate association of the observed behavioral anomaly withs Th17-cell repertoire since Th1-cell population remained unaffected on DG treatment.

### Th17 cells infiltrate regions that regulate grooming activity and are implicated in the pathophysiology of OCD

**Figure 5:**
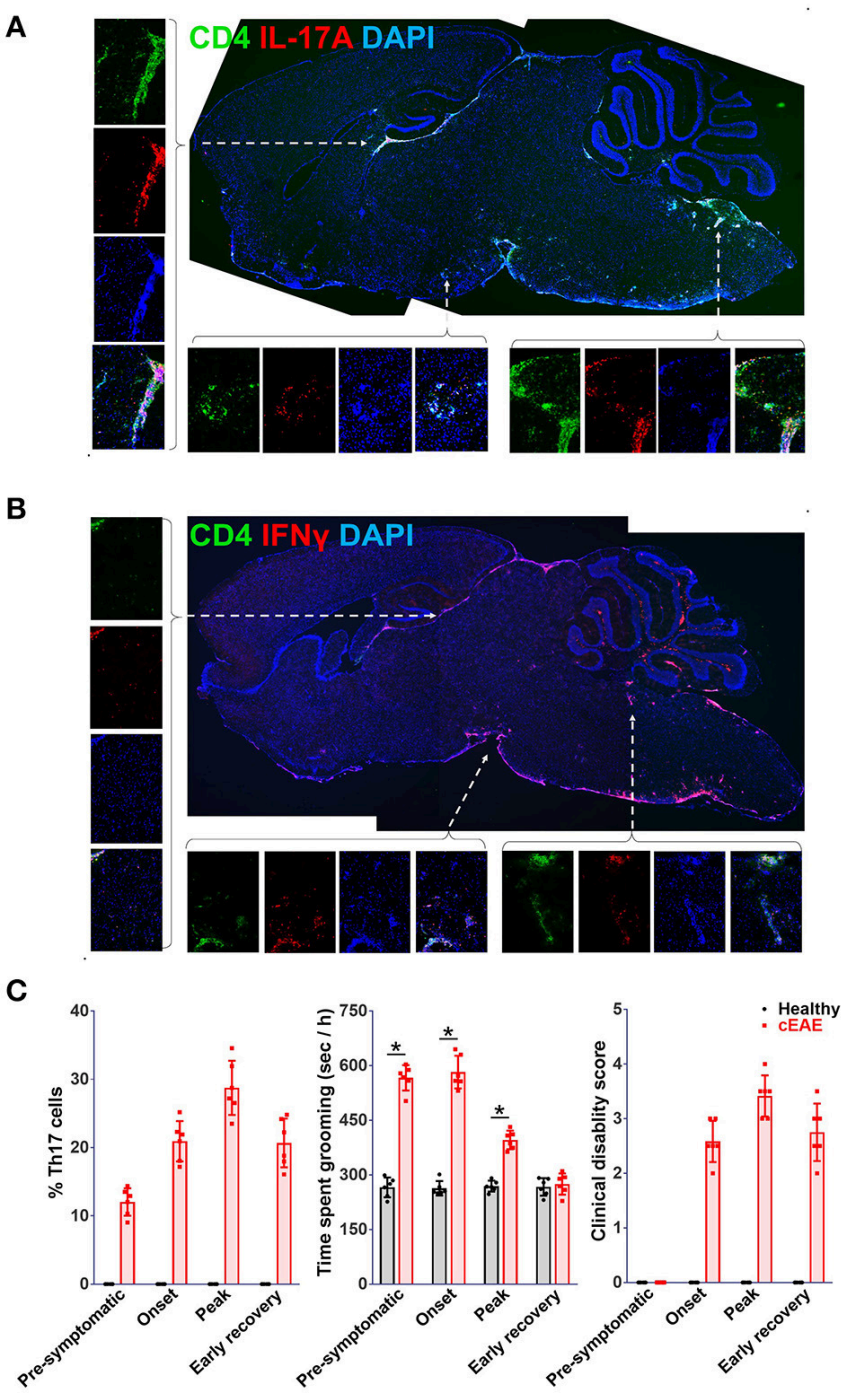
Th17-cells infiltrate brain regions implicated in pathophysiology of obsessive-compulsive disorder. Cryo-sections of brain-tissue (saggital), derived from *Rag1*^−/−^ mice transferred with **(A)** Th17, **(B)** Th1-cells (*2D2* derived) were stained for IL-17A or IFNγ, representative confocal photomicrographs. Images on the right provide enlarged view of the area marked i.e. brain stem (Bs) and cortex (Cx). For correlation analysis mice with cEAE were monitored for the presence of Th17-cells in brain, compulsive-grooming and severity of cEAE, **(C)** frequencies of Th17-cells in brain, severity of compulsive grooming (time spent grooming) and clinical disability recorded on day 10 (pre-symptomatic), 15 (onset), 20 (peak) and 25 (early recovery). mean ± S.D., *n* = 6, ^*^p < 0.05.

The brain-tissue of *Rag1*^−/−^mice transferred with MOG (35-55) reactive Th1 or Th17 were analyzed for cellular infiltration. Th1 or Th17 cells transferred adoptively were found to be scattered throughout the brain parenchyma. In the brain-tissue of the mice grooming excessively (transferred with Th17 cells), Th17-cells were found to be lodged primarily in brainstem and cortex, regions known to regulate grooming in mice and dysregulation of which also coincides with OCD (50–52) (Figure 5A). Whereas, the mice with prominence of Th1 cells (transferred with Th1 cells) in these regions had normal grooming activity (Figure 5B). Further, mice immunized with MOG (35-55) were monitored individually at various stages of EAE (day 10, pre-symptomatic; day 15, onset; day 20, peak; and day 25, early recovery) to explore how the severity of behavioral phenotype relates with frequencies of Th17-cells in the brain or clinical disability. The frequencies of Th17-cells in the brain increased with EAE severity and peaked simultaneously with clinical disability score till peak of the disease, which was in line with a study by Korn et al. (53). The severity of compulsive behavior was found to be correlating with Th17 cell numbers. The grooming reflex dwindled away with further increment in clinical disability during the later stages of the disease (clinical score >2) (Figure 5C). This could be attributed to acute morbidity and possibly to the widespread neuronal damage in the regions regulating grooming reflexes. These findings further consolidated the contention that Th17-cells were orchestrating the malfunctioning of the neuronal circuit controlling grooming behavior.

### Serotonin-reuptake inhibitor ameliorates behavioral pathology

**Figure 6:**
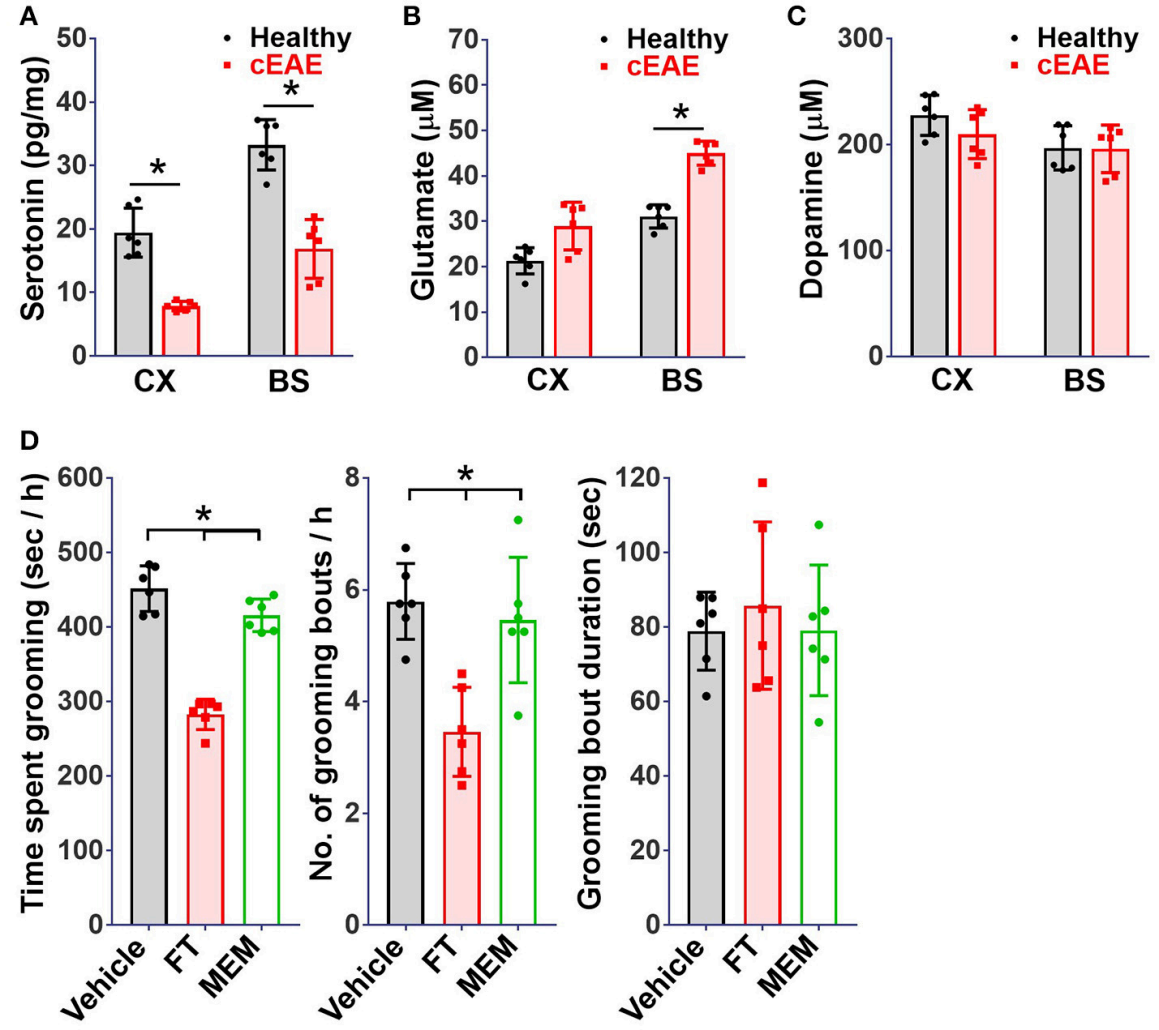
Serotonin re-uptake inhibitor ameliorates behavioral pathology. Concentrations of **(A)** serotonin, **(B)** glutamate, **(C)** dopamine, in brain-tissue homogenates (cortical (Cx) and brainstem (Bs) regions) of mice with cEAE at day 10 post-immunization (*n* = 6). **(D)** Grooming analysis *viz*. time spent grooming, number and duration of grooming bouts, when injected (intraperitoneally) with fluoxetine (FT, selective serotonin reuptake inhibitor, 5 mg/kg body weight) or memantine (MEM, NMDAR antagonist, 15 mg/kg body weight) from day 4-9 (*n* = 6). Mean ± S.D., ^*^p < 0.05.

Among various neuro-transmitters, the involvement of serotonin (54), glutamate (55), and dopamine (56) has been well documented in the patho-physiology of OCD. The levels of serotonin, glutamate and dopamine were analyzed in brain-tissue homogenates of diseased mice. While the levels of dopamine remained comparable, a considerable reduction in serotonin levels was noted both in brain stem and cortex (Figures 6A-C). Besides changes in serotonin levels, glutamate levels were elevated particularly in brain-stem region. Next, we treated diseased mice with fluoxetine (FT, a serotonin reuptake inhibitor, SSRI) or memantine (MEM, an un-competitive inhibitor of N-methyl-D-aspartate receptor (57). FT treated mice showed considerable remission in grooming pathology while effect of MEM was marginal (Figure 6D). The serotonin re-uptake inhibitors constitute first line treatment option for OCD (58). The beneficial effect of FT in this case in a way ascertains its resemblance to OCD.

## Discussion

There exists a fundamental link between physical and mental health. Any perturbations in one can have adverse effects on the other. Autoimmune diseases are generally chronic and refractory to treatment. As a result, patients with such disorders are exposed to very high levels of psychological stress which in many cases has been reported to culminate into serious neuro-psychiatric conditions. Given the predominance of aberrant immune responses in such disorders it is equally likely that immune cells may be modulating behavioral outcomes in such patients.

In the present study, we examined the routine behavior of mice affected with chronic experimental autoimmune encephalomyelitis in order to assess the psychological impact of the disorder. We observed unexpectedly high grooming activities in diseased mice which in some cases manifested as hair-less patches and/or injuries. The repetitive behavior was noted to be quite similar to OCD in human subjects in the light of following evidences: firstly, diseased mice devoted unusually greater time in grooming themselves which can be viewed as over-grooming; second, the grooming behavior was rigid in pattern and rigidity is a characteristic feature of OCD; thirdly, the behavior had a anxiety component as revealed by a marked increase in marble burying and nestlet shredding activity which is again a hallmark of OCD (59, 60); fourthly, the behavioral anomaly was relieved by SSRI treatment indicating perturbations in the serotonergic neurotransmission which is mostly implicated in the pathogenesis of OCD.

We also found a direct involvement of Th17 cells in triggering the repetitive behavior. The involvement was first suspected upon analysis of brain infiltrating mononuclear cells which revealed Th17 numbers to be markedly increased in the brain of affected mice. Since autoimmune disorders are characterized by both aberrant Th17 and Th1 responses it was important to delineate their specific roles. We performed adoptive transfer of Th17 and Th1 cells derived from *2D2* mice into *B6.Rag1*^−/−^ mice. To our surprise, EAE developed in both the experimental groups but repetitive behavior was seen only in mice transferred with Th17 cells. We then assessed the development of autoimmunity in *IFN*γ^−/−^ mice since these mice are defective in Th1 responses (48). These mice developed both autoimmunity and behavioral anomaly, upon immunization with encephalitogenic peptides, lending further support to our surmise on the involvement of Th17-cells in the observed behavioral pathology. These findings provided direct evidence on the role of Th17 cells as key stimulators of behavioral abnormality. Consequently, selective depletion of Th17 cells using digoxin ameliorated both autoimmune and psychological manifestations of the disorder. It may be mentioned here that besides being a selective Th17 cell inhibitor, digoxin is a well-known cardiac glycoside that inhibits Na^+^/K^+^ ATPase and affects locomotor activity in mice at high concentrations. We have used digoxin at much lower concentration (0.5 mg/kg) which ameliorated grooming compulsions in digoxin treated EAE mice by selectively depleting Th17 cell number.

Grooming activity in MOG(35-55) immunized *C57BL6/J* mice was analyzed throughout the disease i.e. pre-symptomatic (day 10), onset (day 15), peak (day 20), and early recovery (day 25) and significant increase in grooming activity was seen as early as day 10 which continued till day 15 and started to dwindle away post day 20. The severity of compulsive grooming was found to correlate well with Th17 cell numbers in CNS. Unlike MOG (35-55) induced EAE in *C57BL6/J* mice, *SJL/J* mice affected with PLP(131-151) induced EAE did not show significant differences in grooming activities. It may be reasoned that in PLP(131-151) induced EAE, Th17 cells are detectable in the CNS during the peak and early recovery phase of the disease (53). The absence of Th17 cells during early phases of EAE (pre-symptomatic and onset) is likely to underlie absence of grooming pathology in this model. Also, genetic background, antigen specificity of Th17 cells, timing of their infiltration into the CNS may be important contributing factors affecting grooming activity.

Differences in the grooming activities were maximum and comparable at day 10 and day 15 and therefore former was chosen as the preferred time point for subsequent analyses. Further the animals were free from clinical disability at this time point which in our view should be true representation of grooming behavior as physical disability may hamper grooming activity which was indeed noted at later time points (peak and early recovery).

Sensory disturbances and dysesthesia are commonly associated with EAE. The grooming analysis in the present study was largely focused at the pre-symptomatic phase of EAE wherein we did not find any abnormality in response to thermal and mechanical stimuli. Our results are in corroboration with the findings of Jianning et al. (61) who have characterized the sensory abnormalities during the course of EAE in *C57BL6/J* mice and noted hypersensitivity to thermal stimuli only during the chronic phase of the disease and mechanical allodynia during onset and peak. However, the influence of sensory disturbances on grooming activity during the chronic phase of the disease cannot be completely ruled out and (these) might be contributing to self-inflicted injuries noted in a few isolated cases during the chronic phase of EAE.

We further found that fluoxetine, an SSRI, was effective at relieving grooming compulsions indicating disturbances in serotonergic neurotransmission by Th17 cells at the root of the observed compulsive behavior. These alterations in serotonin biology in part may be attributed to the actions of pro-inflammatory cytokines IL-6, IL-1β, TNF-α which are produced upon activation of microglia by Th17 cells. IL-6 has been shown to inhibit the synthesis of serotonin by decreasing levels of tetrahydrobiopterin, an important cofactor (62, 63), TNF-α activates the enzyme indoleamine 2, 3 dioxygenase which breaks down the serotonin precursor, tryptophan, thereby reducing the levels of serotonin in the brain (11, 64). Further, it is also likely that irregularities of other neurotransmitters particularly glutamate may also be involved in the pathogenesis of the observed compulsive behavior as Th17 cells have been reported to establish direct contacts with neurons and produce copious amounts of glutamate upon neuronal interaction (19). Also, the glutamate is the primary excitatory neurotransmitter in the mammalian brain and ~60% of neurons in the brain utilize glutamate as their primary neurotransmitter, perturbations of glutamatergic neurotransmission underlie several neuropsychiatric disorders including OCD. For example, increased levels of glutamate or its metabolites have also been found in the cerebrospinal fluid (CSF) (65), caudate nucleus, orbitofrontal cortex (OFC) and cingulate cortex and their levels correlate with the severity of OCD (66). Pharmacologic inhibitors of glutamatergic neurotransmission such as riluzole, cycloserine and memantine have been successfully used in OCD patients unresponsive to SSRIs (55).

Our observations were further supported by the immuno-histochemical analyses which revealed infiltration of brainstem and cortex by Th17 cells. These regions have been identified to be important for generation of basic elements of a grooming syntax. Hence, modulation of neuronal activity by Th17 cells in these regions may explain frequent initiation of grooming reflexes as seen in cEAE mice. The observed changes in mice behavior were noted to be concomitant with the presence of Th17-cells in the brain stem of diseased mice.

There is a growing body of evidences which directly or indirectly support the potential of Th17 cells to modulate CNS functions. In a recent report by Beurel et al. it has been demonstrated that Th17 cells are capable of inducing depression like behavior in mice (16). Even in schizophrenia, the involvement of Th17 cells in the immuno-pathogenesis has been highlighted (67). Occurrence of Th17 phenotype in Generalized Anxiety Disorder patients (14) and increased production of IL-17 by their T-lymphocytes (15) has also been recently demonstrated. All these reports further underscore the clinical relevance of our study and reinforce an unexplored functionality for this lymphocytic subset. Hence, appropriately it could be proposed that autoimmunity due to Th17-cells or any condition leading to a persistent increase in this particular repertoire of immune cells is a risk factor for neuropsychiatric illnesses.

## Author contributions

RK and AS conceived and designed experiments. AS provided the entire infrastructure and supervised the research. RK and SP performed the experiments. RK, SP, and AS wrote the manuscript. AS corrected the manuscript.

### Conflict of interest statement

The authors declare that the research was conducted in the absence of any commercial or financial relationships that could be construed as a potential conflict of interest.
