# Supplementary material for: Auto-Reactive Th17-Cells Trigger Obsessive-Compulsive-Disorder Like Behavior in Mice With Experimental Autoimmune Encephalomyelitis
Source: Front Immunol. 2018 Oct 31;9:2508. doi: 10.3389/fimmu.2018.02508 (PMC6220041; doi:10.3389/fimmu.2018.02508)
Supplement: Supplementary file 3 [file Image_1.pdf]

SUPPLEMENTARY INFORMATION

Auto-reactive Th17-cells trigger obsessive-compulsive-disorder like behavior in mice with experimental autoimmune encephalomyelitis

Ravi Kant, Shweta Pasi, Avadhesha Surolia

SUPPLEMENTARY FIGURES

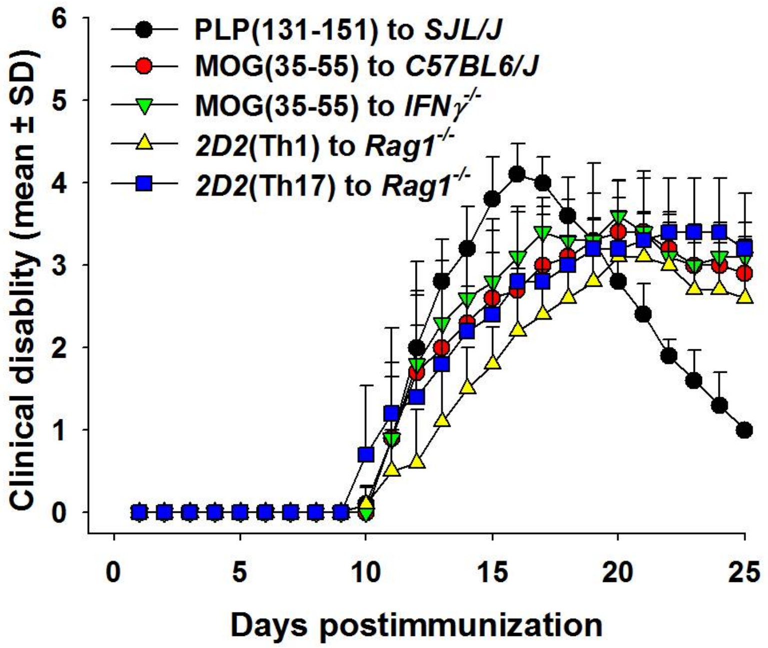

Supplementary Fig. 1 Experimental autoimmune encephalomyelitis (EAE): clinical progression. Relapsing-remitting EAE (rrEAE) was induced in *SJL/J* mice (female, 8-10 week old) by immunization with PLP(131-151). EAE was induced in *C57BL6/J* mice (female, 8-10 week old, *WT* or *IFNγ*$^{-/-}$) by immunization with MOG(35-55). *Rag1*$^{-/-}$ (female, 8-10 week old) transferred adoptively with *2D2* reactive Th1 or Th17 cells. Clinical disability score. mean±S.D., n≥6.

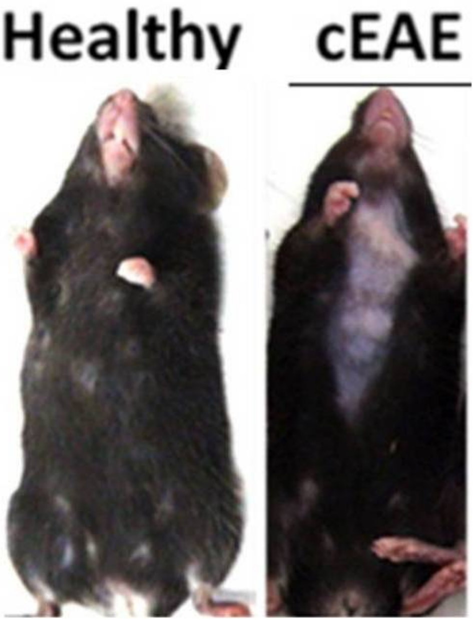

Supplementary Fig. 2 *C57BL6/J* mice (8-10 week old, females) immunized with MOG(35-55), hair-loss phenotype, a representative picture.

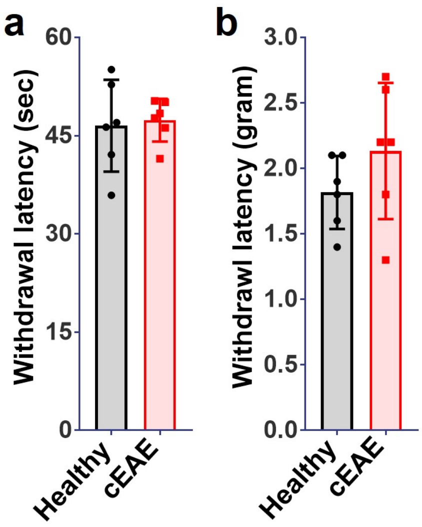

Supplementary Fig. 3 *C57BL6/J* mice (8-10 week old, females) immunized with MOG(35-55) were subjected to **(a)** hot-plate test, (b) van Frey test on day 10 post-immunization, paw withdrawal latency. mean±S.D., n=6.

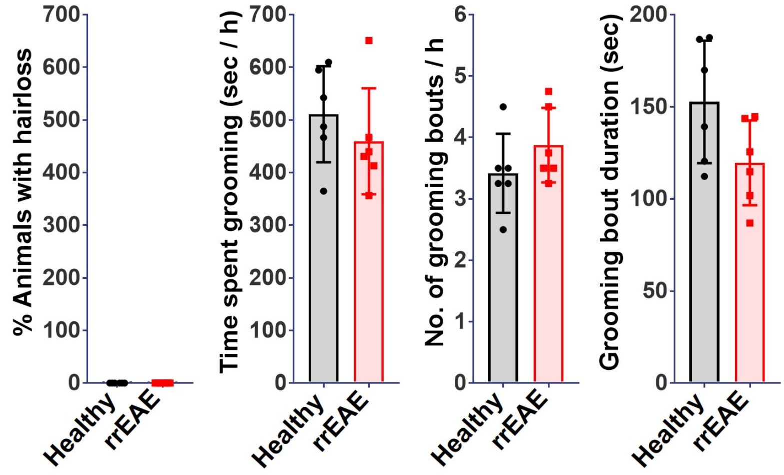

Supplementary Fig. 4 Relapsing-remitting form of EAE (rrEAE) was induced in *SJL/J* (female, 8-10 week) by immunization with PLP(131-151), grooming analysis *viz.* time spent grooming, number of grooming bouts, duration of grooming bouts (on day 10). mean±S.D., n=6.

Supplementary Movie 1 Grooming activity of healthy *C57BL/6J* mice, related to Figure 1
The video shows grooming activity in a normal *C57BL/6J* mice.

Supplementary Movie 2 Grooming activity of *C57BL/6J* mice affected with cEAE, related to Figure 1
The movie shows video-recording of cEAE-mice on day 10 post immunization with MOG (35-55). The mice can be seen performing excessive facial cleaning along with body licking.
